# Novel Reassortant Highly Pathogenic Avian Influenza A(H5N2) Virus in Broiler Chickens, Egypt

**DOI:** 10.3201/eid2601.190570

**Published:** 2020-01

**Authors:** Kareem E. Hassan, Jacqueline King, Magdy El-Kady, Manal Afifi, Hassanein H. Abozeid, Anne Pohlmann, Martin Beer, Timm Harder

**Affiliations:** Friedrich-Loeffler-Institut, Greifswald-Riems, Germany (K.E. Hassan, J. King, A. Pohlmann, M. Beer, T. Harder);; Beni Suef University, Beni Suef, Egypt (K.E. Hassan, M. El-Kady);; Cairo University, Cairo, Egypt (M. Afifi, H.H. Abozeid)

**Keywords:** avian influenza virus, zoonoses, reassortant, viruses, respiratory infections, H5N2, poultry, Egypt, influenza, broiler chickens, H5N2, highly pathogenic avian influenza

## Abstract

We detected a novel reassortant highly pathogenic avian influenza A(H5N2) virus in 3 poultry farms in Egypt. The virus carried genome segments of a pigeon H9N2 influenza virus detected in 2014, a nucleoprotein segment of contemporary chicken H9N2 viruses from Egypt, and hemagglutinin derived from the 2.3.4.4b H5N8 virus clade.

Since 2006, Egypt’s poultry industry has been plagued by endemic infections with highly pathogenic avian influenza (HPAI) virus, subtype H5N1, clade 2.2.1, of the goose/Guangdong (gs/GD) lineage (*1*). In addition, low pathogenicity avian influenza (LPAI) virus of subtype H9N2, G1 lineage, introduced in 2011 (*2*), and HPAI H5N8 (gs/GD clade 2.3.4.4b) introduced in 2016, have become entrenched in local poultry populations (*3*). Despite ongoing control measures, respiratory disease with increased mortality rates is endemic in poultry farms in Egypt.

The zoonotic nature of HPAI H5N1 2.2.1 viruses has caused in Egypt the highest number of human infection cases per country worldwide; a low level of sporadic benign human cases of H9N2 viral infection has also been reported from Egypt (*4*). Continued adaptation by point mutations, but not reassortment, to enhance replication in mammalian hosts has been repeatedly reported in avian influenza in Egypt (*5*). Here, we describe the detection of a new reassortant HPAI virus in commercial chicken holdings in Egypt. This virus carries the hemagglutinin (HA) gene of HPAI clade 2.3.4.4b H5N8 virus and 7 genome segments derived from Egyptian H9N2 viruses (*6*).

## The Study

During January–April 2019, we examined samples from 11 commercial broiler farms reporting respiratory clinical signs among chickens by using the Riems Influenza A Typing Assay (*7*). We detected co-presence of avian influenza viruses subtypes H5 and H9 with N2 (8 farms) as well as H5N2 only (3 farms).

**Figure 1:**
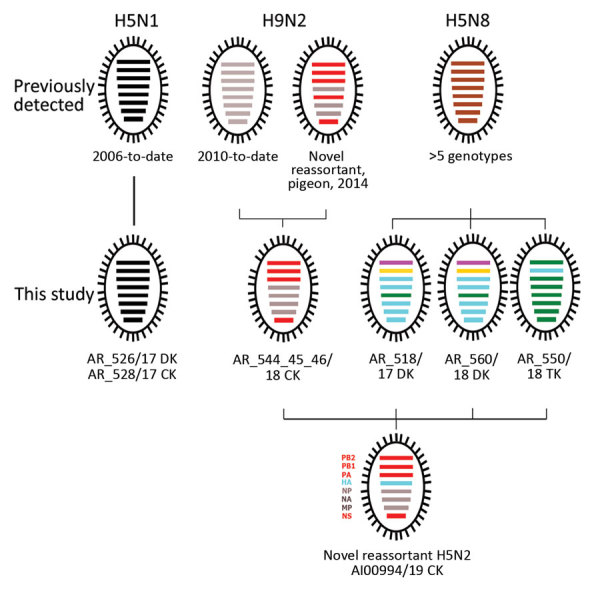
Genotype and reassortment analyses based on full-length genome sequences of avian influenza viruses in Egypt previously detected and those identified in this study. Colors indicate grouping of segment origin according to phylogenetic analyses (Appendix 1 Figure): highly pathogenic avian influenza (HPAI) H5N1 2.2.1.2 virus from Egypt (black); H9N2 subtype from Egypt circulating in chickens since 2010 (gray); H9N2 subtype from Egypt first detected in pigeons in 2014 (red); HPAI H5N8 viruses previously detected and circulating in Egypt (brown; different genotypes); Polymerase basic (PB) 2 segment most closely related to an H3N6 virus from Bangladesh (purple); PB1 segment most closely related to an H7N7 virus from Georgia (yellow); segments most closely related to H5N8 viruses from China (blue) or Russia (green).

**Figure 2:**
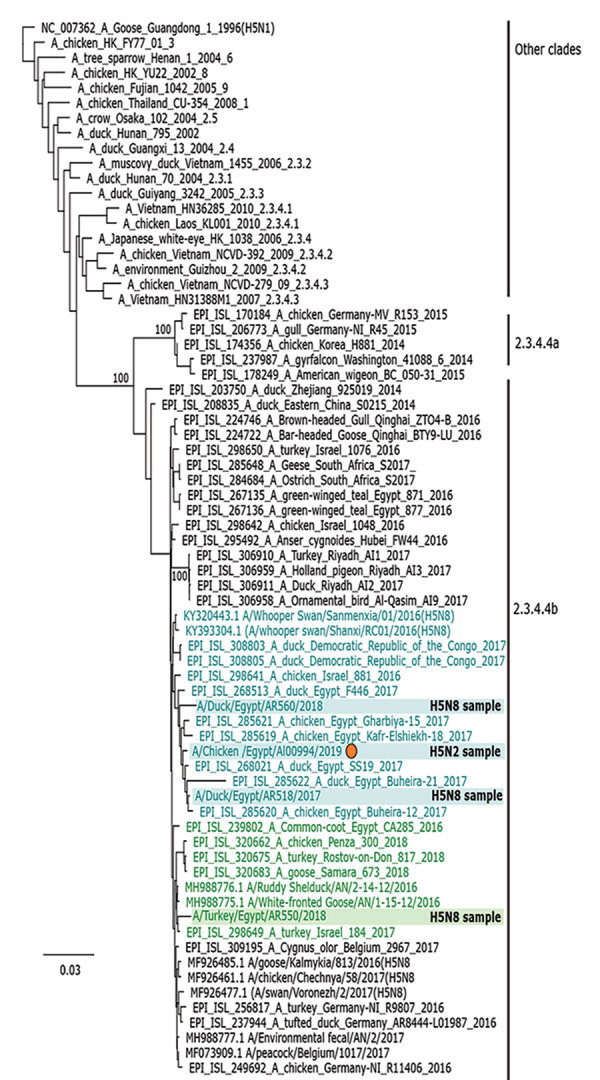
Phylogenetic analysis of the hemagglutinin segments of reassortant highly pathogenic avian influenza H5N2 and H5N8 viruses belonging to clade 2.3.3.4b from Egypt and reference viruses. Sequence analysis was based on alignment analyses by MAFFT version 7.450 embedded in the Geneious software suite, version 11.1.7 (https://www.geneious.com) with manual editing. We performed maximum-likelihood calculations using PhyML version 3.0 (http://www.atgc-montpellier.fr/phyml); we chose the best-fit model according to the Bayesian selection criterion using Model Finder embedded in Geneious. Colors indicate grouping of segment origin and match those shown in Figure 1: blue, most closely related to H5N8 viruses from China; green, most closely related to H5N8 viruses from Russia and Europe. GenBank or GISAID accession numbers (http://www.gisaid.org) are provided for reference sequences.

We selected 8 samples representing H5N8, H9N2, and H5N1 from 2017–2018, plus 1 positive H5N2 sample from 2019, for full-genome sequencing (Table 1; Appendix 1 Table). Sanger- and next-generation sequencing results identified various reassortants new to Egypt (Figure 1). All H5 HA segments encoded a polybasic cleavage site, PLREKRRKR-GLF (H5 clade 2.3.4.4b) or PQGEKRRKKR-GLF (H5 clade 2.2.1.2), thus classifying those viruses as highly pathogenic. We identified the closest related sequences by BLAST (https://blast.ncbi.nlm.nih.gov/Blast.cgi) homology searches in the GISAID (http://platform.gisaid.org) and International Nucleotide Sequence Data Collaboration (http://www.insdc.org) databases. Phylogenetic analyses of each genome segment aided in clustering sequences (Appendix 1 Figure). We delineated the putative origin of each of the genome segments (Figure 1). The HA segment of the novel HPAI H5N2 reassortant virus was derived from clade 2.3.4.4b viruses with closest homology to viruses circulating in ducks in Egypt in 2017 (Figure 2), whereas >4 additional genome segments (polymerase basic 1, polymerase basic 2, polymerase, and nonstructural protein) originated from novel reassortant H9N2 viruses first detected in pigeons in Egypt during 2014 (*6*). The nucleoprotein segment and perhaps others were acquired from H9N2 viruses circulating in chickens in Egypt since 2010. Matrix and neuraminidase segments are identical in the pigeon and chicken H9N2 viruses. We identified no new mutations in the genome of reassortant H5N2 that would suggest increased adaptation to mammalian hosts. In addition, we observed 2 previously undescribed genotypes of HPAI H5N8 with distinct polymerase basic 1 and 2 segment origins (Figure 1). The composition of HPAI H5N1 viruses phylogenetically assigned to clade 2.2.1.2 (Figure 1; Appendix 1 Figure) was unaltered compared with other HPAI H5N1 viruses isolated since 2015.

**Table:** Characteristics of avian influenza viruses in samples from diseased poultry, Egypt*

Strain	Subtype	Collection date	Host species	Governorate	Flock size	Mortality rate, %	Genome sequence	Clade
A/duck/Egypt/AR518/2017	H5N8	2017 Mar 15	Duck	Giza	3,000	40	Full	2.3.4.4b
A/duck/Egypt/AR560/2018	H5N8	2018 May 20	Duck	Giza	5,000	35	Full	2.3.4.4b
A/turkey/Egypt/AR550/2018	H5N8	2018 Mar 2	Turkey	Beni-Suef	5,000	100	Full	2.3.4.4b
A/duck/Egypt/AR526/2017	H5N1	2017 Mar 20	Duck	Beni-Suef	3,000	15	Full	2.2.1.2
A/chicken/Egypt/AR528/2017	H5N1	2017 Mar 22	Chicken, layer	Beni-Suef	5,000	30	Full	2.2.1.2
A/chicken/Egypt/AR544/2018	H9N2	2018 Jan 20	Chicken, broiler	Giza	10,000	25	Full	G1.B
A/chicken/Egypt/AR545/2018	H9N2	2018 Mar 25	Chicken, broiler	Qualiobia	5,000	25	Full	G1.B
A/chicken/Egypt/AR546/2018	H9N2	2018 Mar 22	Chicken, broiler	El-Menia	8,000	10	Full	G1.B
A/chicken/Egypt/Al00994/2019	H5N2	2019 Jan 19	Chicken, broiler	Beheira	17,000	47	Full	2.3.4.4b
A/chicken/Egypt/AI00986/2019	H5N2	2019 Jan 5	Chicken, broiler	Fayoum	10,000	5	HA (partial)	2.3.4.4b
A/chicken/Egypt/AI00987/2019	H5, H9, N2	2019 Jan 9	Chicken, broiler	Beheira	7,000	15	HA (partial)	2.3.4.4b
A/chicken/Egypt/AI00988/2019	H5, H9, N2	2019 Jan 19	Chicken, broiler	Beheira	8,000	20	HA (partial)	2.3.4.4b
A/chicken/Egypt/AI00989/2019	H5, H9, N2	2019 Jan 27	Chicken, broiler	El-Menia	6,000	14	HA (partial)	2.3.4.4b
A/chicken/Egypt/AI00991/2019	H5, H9, N2	2019 Feb 16	Chicken, broiler	Beni-Suef	74,000	16	NA*	NA
A/chicken/Egypt/AI00992/2019	H5N2	2019 Mar 3	Chicken, broiler	Beheira	5,000	15	HA (partial)	2.3.4.4b
A/duck/Egypt/AI00993/2019	H5, N8, N2	2019 Jan 14	Chicken, broiler	Giza	4,000	15	NA	NA
A/chicken/Egypt/AI00995/2019	H5, H9, N2	2019 Jan 14	Chicken, broiler	Beheira	33,000	33.3	NA	NA
A/chicken/Egypt/AI00996/2019	H5,H9, N2	2019 Jan 15	Chicken, broiler	Beheira	10,000	60	NA	NA
A/chicken/Egypt/AI00997/2019	H5, N8, H9, N2	2019 Mar 9	Chicken, broiler	Dakhalia	40,000	7.5	NA	NA

*HA, hemagglutinin; NA, not available.

Natural reassortants between H5 HPAI of the gs/GD lineage and H9N2 viruses, including subtype H5N2, have repeatedly emerged in Southeast Asia (*8*). So far, both the HPAI H5N1 2.2.1.2 and the co-circulating H9N2-G1 viruses appeared to be genotypically stable in poultry in Egypt. Successful forced reassortment of these viruses by co-cultivation and serum selection in embryonated chicken eggs ruled out a principal incompatibility between their genome segments (*5*); however, Naguib et al. did not rescue an H5N2 reassortant.

We and others have shown that HPAI H5 viruses of clade 2.3.4.4 have a high tendency to reassort with various influenza A viruses of wild birds or poultry (*9*). Thus, the incursion of clade 2.3.4.4b viruses into Egypt in 2016 not only added another antigenically distinct HPAI virus, but also signaled an increased reassortment risk. In fact, the 2.3.4.4b H5N8 virus proved to be a parent of the newly emerged H5N2 reassortant. Likewise, the second parental virus, an influenza A(H9N2) virus first detected in pigeons, was not described in Egypt before 2014. Genotypically, this H9N2 virus is distinct from the third parental virus, that is, the original H9N2 virus introduced to poultry in Egypt in 2010 (Figure 1). Infection of pigeons with clade 2.3.4.4b H5N8 HPAI virus has been described in Egypt, although pigeons are believed to be less susceptible to avian influenza infections (*10*). Although we cannot attribute the origin of the current HPAI H5N2 reassortant to a single host species, we cannot exclude pigeons as a possible host.

In March 2019, Egypt’s Ministry of Agriculture announced the detection of a new influenza A(H5N2) virus from seemingly healthy ducks in the Dakahlia governorate *(**11*); recently published information on this reassortant indicated the presence of a neuraminidase N2 segment of chicken H9N2 viruses in the background of an HPAI H5 clade 2.3.4.4b virus (*11*). Our data confirm the presence of a different H5N2 reassortant and its occurrence in chickens in different geographic regions of Egypt (Table). We detected the current reassortant HPAI H5N2 viruses in 2 different broiler farms in Beheira (January and March 2019) and 1 broiler farm in Fayoum (January 2019) governorates (Table). The HA amino acid sequence of these reassortants does not signal antigenic variation compared with parent HPAI H5 subtype of clade 2.3.4.4b. Antigenic and further phenotypic properties, such as host specificity, require investigation as soon as isolates are available. For the H5N2-positive samples, only FTA card material was available at Friedrich-Loeffler-Institut. However, H5N2 isolates were successfully generated at the Beni-Suef University, Egypt, but were currently not available for further antigenic and phenotypic analyses (M. El-Kady, unpub. data).

Intensified targeted surveillance in poultry and pigeons is urgently required and may lead to detection of additional reassortants. However, co-detection in a sample of H5N8 and H9N2 subtypes by reverse transcription quantitative PCR may blur the identification of H5N2 reassortants; plaque purification of such samples would aid in separating subtypes but cannot currently be used in routine diagnostics.

There is a risk for transboundary spread of HPAI A(H5N2) virus in northern Africa and the Middle East, and similar reassortment events are to be expected in regions where clade 2.3.4.4 HPAI and H9N2 viruses are co-circulating. Long-term solutions in combating avian influenza virus infections in poultry are sorely needed and would help to lower risks of human exposure to zoonotic avian influenza viruses such as the highly zoonotic H7N9 viruses in China that carry a full set of internal genes of an H9N2-G1–like avian influenza virus (*12*).

Appendix 1 Additional information about highly pathogenic avian influenza A(H5N2) virus in broiler chickens, Egypt.

Appendix 2 List of reference sequences from GISAID (http://www.gisaid.org) used in study of highly pathogenic avian influenza A(H5N2) virus in broiler chickens, Egypt.
