# Supplementary material for: Novel Reassortant Highly Pathogenic Avian Influenza A(H5N2) Virus in Broiler Chickens, Egypt
Source: Emerg Infect Dis. 2020 Jan;26(1):129–33. doi: 10.3201/eid2601.190570 (PMC6924912; doi:10.3201/eid2601.190570)
Supplement: Appendix 1 — Additional information about highly pathogenic avian influenza A(H5N2) virus in broiler chickens, Egypt. [file 19-0570-Techapp-s1.pdf]

Article DOI: https://doi.org/10.3201/eid2601.190570

Novel Reassortant Highly Pathogenic Avian Influenza A(H5N2) Virus in Broiler Chickens, Egypt

Appendix 1

Appendix 1 Table. Avian influenza sequences included in study of highly pathogenic avian influenza virus A(H5N2), Egypt

AIV designation	AIV subtype	GISAID sequence accession no.							
		PB2	PB1	PA	HA	NP	NA	M	NS
A/Chicken/Egypt/AI00994/2019	H5N2	EPI1420452	EPI1420451	EPI1420416	EPI14204383	EPI1420434	EPI1420450	EPI1420384	EPI1420397
A/Duck/Egypt/AR518/2017	H5N8	EPI1420351	EPI1420350	EPI1420349	EPI1381391	EPI1420346	EPI1420345	EPI1420344	EPI1420347
A/Duck/Egypt/AR560/2018	H5N8	EPI1420334	EPI1420333	EPI1420332	EPI1381402	EPI1420330	EPI1420329	EPI1420328	EPI1420331
A/Turkey/Egypt/AR550/2018	H5N8	EPI1420343	EPI1420342	EPI1420341	EPI1381395	EPI1420339	EPI1420338	EPI1420336	EPI1420340
A/Duck/Egypt/AR526/2017	H5N1	EPI1420366	EPI1420364	EPI1420363	EPI1381393	EPI1420358	EPI1420357	EPI1420355	EPI1420362
A/Chicken/Egypt/AR528/2017	H5N1	EPI1420377	EPI1420376	EPI1420375	EPI1381432	EPI1420372	EPI14203571	EPI1420369	EPI1420373
A/Chicken/Egypt/AR544/2018	H9N2	EPI1420308	EPI1420307	EPI1420306	EPI1381412	EPI1420304	EPI1381411	EPI14203530<br>1	EPI1420305
A/Chicken/Egypt/AR545/2018	H9N2	EPI1420327	EPI1420326	EPI1420325	EPI131413	EPI1420323	EPI14203322	EPI1420321	EPI1420324
A/Chicken/Egypt/AR546/2018	H9N2	EPI1420318	EPI1420317	EPI1420316	EPI1381414	EPI1420314	EPI1420313	EPI1420312	EPI1420315
A/Chicken/Egypt/AI00986/2019	**H5**N2				EPI1542792				
A/Chicken/Egypt/AI00987/2019	**H5**, H9,<br>N2				EPI1542793				
A/Chicken/Egypt/AI00988/2019	**H5**, H9,<br>N2				EPI1542794				
A/Chicken/Egypt/AI00989/2019	**H5**, H9,<br>N2				EPI1542795				
A/Chicken/Egypt/AI00990/2019	**H5**, H9,<br>N2				EPI1542796				
A/Chicken/Egypt/AI00992/2019	**H5**N2				EPI1542798				

*Bold text indicates the hemagglutinin subtype sequenced in samples in which >1 HA subtype was detected. AIV, Avian influenza virus; HA, hemagglutinin, M, matrix; NA, neuraminidase; NP, nucleoprotein; NSP, nonstructural protein; PA, polymerase; PB, polymerase basic.

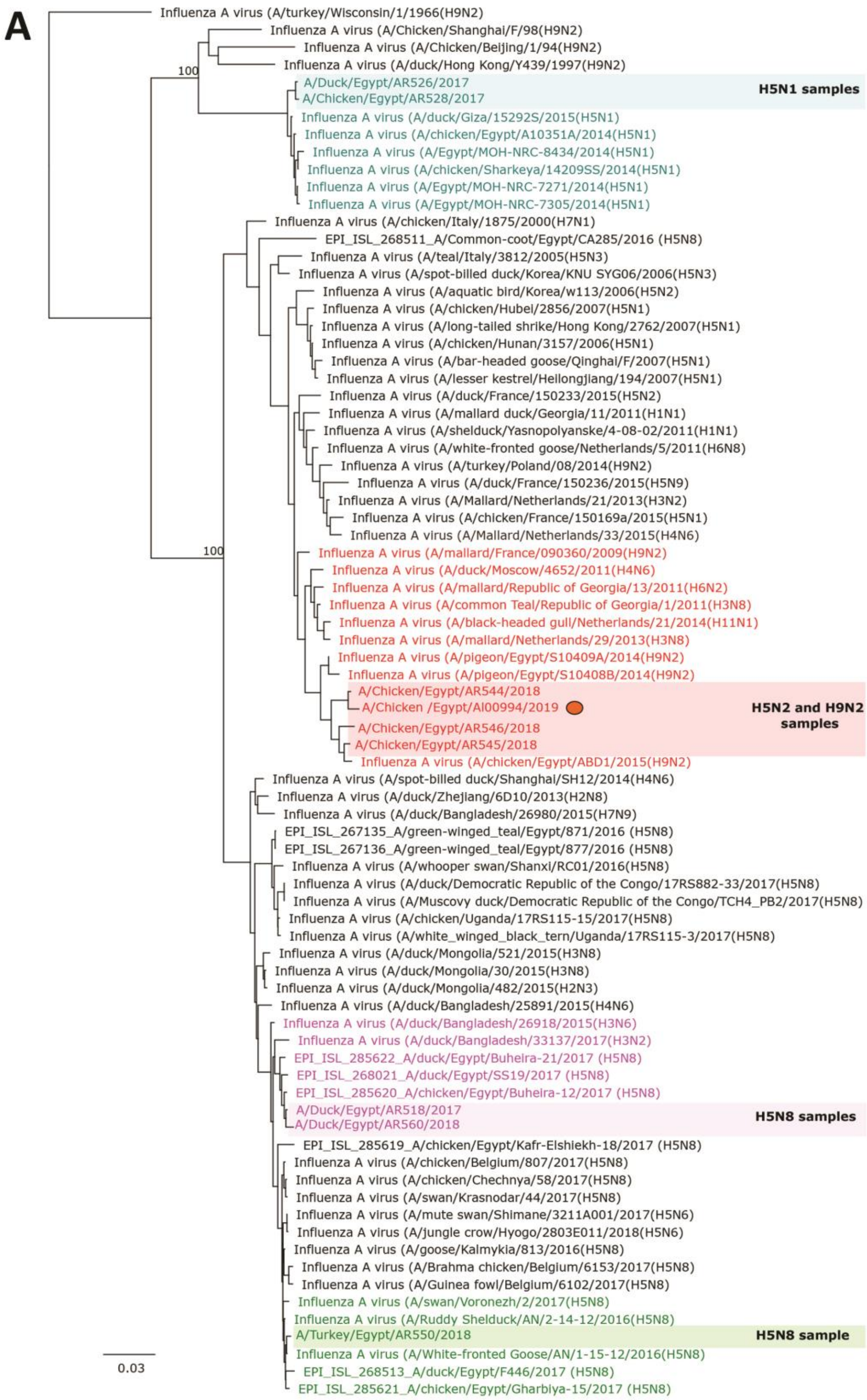
A
Influenza A virus (A/turkey/Wisconsin/1/1966(H9N2)
Influenza A virus (A/Chicken/Shanghai/F/98(H9N2)
Influenza A virus (A/Chicken/Beijing/1/94(H9N2)
Influenza A virus (A/duck/Hong Kong/Y439/1997(H9N2)
100
A/Duck/Egypt/AR526/2017
A/Chicken/Egypt/AR528/2017
H5N1 samples
Influenza A virus (A/duck/Giza/15292S/2015(H5N1)
Influenza A virus (A/chicken/Egypt/A10351A/2014(H5N1)
Influenza A virus (A/Egypt/MOH-NRC-8434/2014(H5N1)
Influenza A virus (A/chicken/Sharkeya/14209SS/2014(H5N1)
Influenza A virus (A/Egypt/MOH-NRC-7271/2014(H5N1)
Influenza A virus (A/Egypt/MOH-NRC-7305/2014(H5N1)
Influenza A virus (A/chicken/Italy/1875/2000(H7N1)
EPI_ISL_268511_A/Common-coot/Egypt/CA285/2016 (H5N8)
Influenza A virus (A/teal/Italy/3812/2005(H5N3)
Influenza A virus (A/spot-billed duck/Korea/KNU SYG06/2006(H5N3)
Influenza A virus (A/aquatic bird/Korea/w113/2006(H5N2)
Influenza A virus (A/chicken/Hubei/2856/2007(H5N1)
Influenza A virus (A/long-tailed shrike/Hong Kong/2762/2007(H5N1)
Influenza A virus (A/chicken/Hunan/3157/2006(H5N1)
Influenza A virus (A/bar-headed goose/Qinghai/F/2007(H5N1)
Influenza A virus (A/lesser kestrel/Heilongjiang/194/2007(H5N1)
Influenza A virus (A/duck/France/150233/2015(H5N2)
Influenza A virus (A/mallard duck/Georgia/11/2011(H1N1)
Influenza A virus (A/shelduck/Yasnopolyanske/4-08-02/2011(H1N1)
Influenza A virus (A/white-fronted goose/Netherlands/5/2011(H6N8)
Influenza A virus (A/turkey/Poland/08/2014(H9N2)
Influenza A virus (A/duck/France/150236/2015(H5N9)
Influenza A virus (A/Mallard/Netherlands/21/2013(H3N2)
Influenza A virus (A/chicken/France/150169a/2015(H5N1)
Influenza A virus (A/Mallard/Netherlands/33/2015(H4N6)
100
Influenza A virus (A/mallard/France/090360/2009(H9N2)
Influenza A virus (A/duck/Moscow/4652/2011(H4N6)
Influenza A virus (A/mallard/Republic of Georgia/13/2011(H6N2)
Influenza A virus (A/common Teal/Republic of Georgia/1/2011(H3N8)
Influenza A virus (A/black-headed gull/Netherlands/21/2014(H11N1)
Influenza A virus (A/mallard/Netherlands/29/2013(H3N8)
Influenza A virus (A/pigeon/Egypt/S10409A/2014(H9N2)
Influenza A virus (A/pigeon/Egypt/S10408B/2014(H9N2)
A/Chicken/Egypt/AR544/2018
A/Chicken /Egypt/AI00994/2019
A/Chicken/Egypt/AR546/2018
A/Chicken/Egypt/AR545/2018
H5N2 and H9N2 samples
Influenza A virus (A/chicken/Egypt/ABD1/2015(H9N2)
Influenza A virus (A/spot-billed duck/Shanghai/SH12/2014(H4N6)
Influenza A virus (A/duck/Zhejiang/6D10/2013(H2N8)
Influenza A virus (A/duck/Bangladesh/26980/2015(H7N9)
EPI_ISL_267135_A/green-winged_teal/Egypt/871/2016 (H5N8)
EPI_ISL_267136_A/green-winged_teal/Egypt/877/2016 (H5N8)
Influenza A virus (A/whooper swan/Shanxi/RC01/2016(H5N8)
Influenza A virus (A/duck/Democratic Republic of the Congo/17RS882-33/2017(H5N8)
Influenza A virus (A/Muscovy duck/Democratic Republic of the Congo/TCH4_PB2/2017(H5N8)
Influenza A virus (A/chicken/Uganda/17RS115-15/2017(H5N8)
Influenza A virus (A/white_winged_black_tern/Uganda/17RS115-3/2017(H5N8)
Influenza A virus (A/duck/Mongolia/521/2015(H3N8)
Influenza A virus (A/duck/Mongolia/30/2015(H3N8)
Influenza A virus (A/duck/Mongolia/482/2015(H2N3)
Influenza A virus (A/duck/Bangladesh/25891/2015(H4N6)
Influenza A virus (A/duck/Bangladesh/26918/2015(H3N6)
Influenza A virus (A/duck/Bangladesh/33137/2017(H3N2)
EPI_ISL_285622_A/duck/Egypt/Buheira-21/2017 (H5N8)
EPI_ISL_268021_A/duck/Egypt/SS19/2017 (H5N8)
EPI_ISL_285620_A/chicken/Egypt/Buheira-12/2017 (H5N8)
A/Duck/Egypt/AR518/2017
A/Duck/Egypt/AR560/2018
H5N8 samples
EPI_ISL_285619_A/chicken/Egypt/Kafr-Elshiekh-18/2017 (H5N8)
Influenza A virus (A/chicken/Belgium/807/2017(H5N8)
Influenza A virus (A/chicken/Chechnya/58/2017(H5N8)
Influenza A virus (A/swan/Krasnodar/44/2017(H5N8)
Influenza A virus (A/mute swan/Shimane/3211A001/2017(H5N6)
Influenza A virus (A/jungle crow/Hyogo/2803E011/2018(H5N6)
Influenza A virus (A/goose/Kalmykia/813/2016(H5N8)
Influenza A virus (A/Brahma chicken/Belgium/6153/2017(H5N8)
Influenza A virus (A/Guinea fowl/Belgium/6102/2017(H5N8)
Influenza A virus (A/swan/Voronezh/2/2017(H5N8)
Influenza A virus (A/Ruddy Shelduck/AN/2-14-12/2016(H5N8)
A/Turkey/Egypt/AR550/2018
H5N8 sample
Influenza A virus (A/White-fronted Goose/AN/1-15-12/2016(H5N8)
EPI_ISL_268513_A/duck/Egypt/F446/2017 (H5N8)
EPI_ISL_285621_A/chicken/Egypt/Gharbiya-15/2017 (H5N8)
0.03

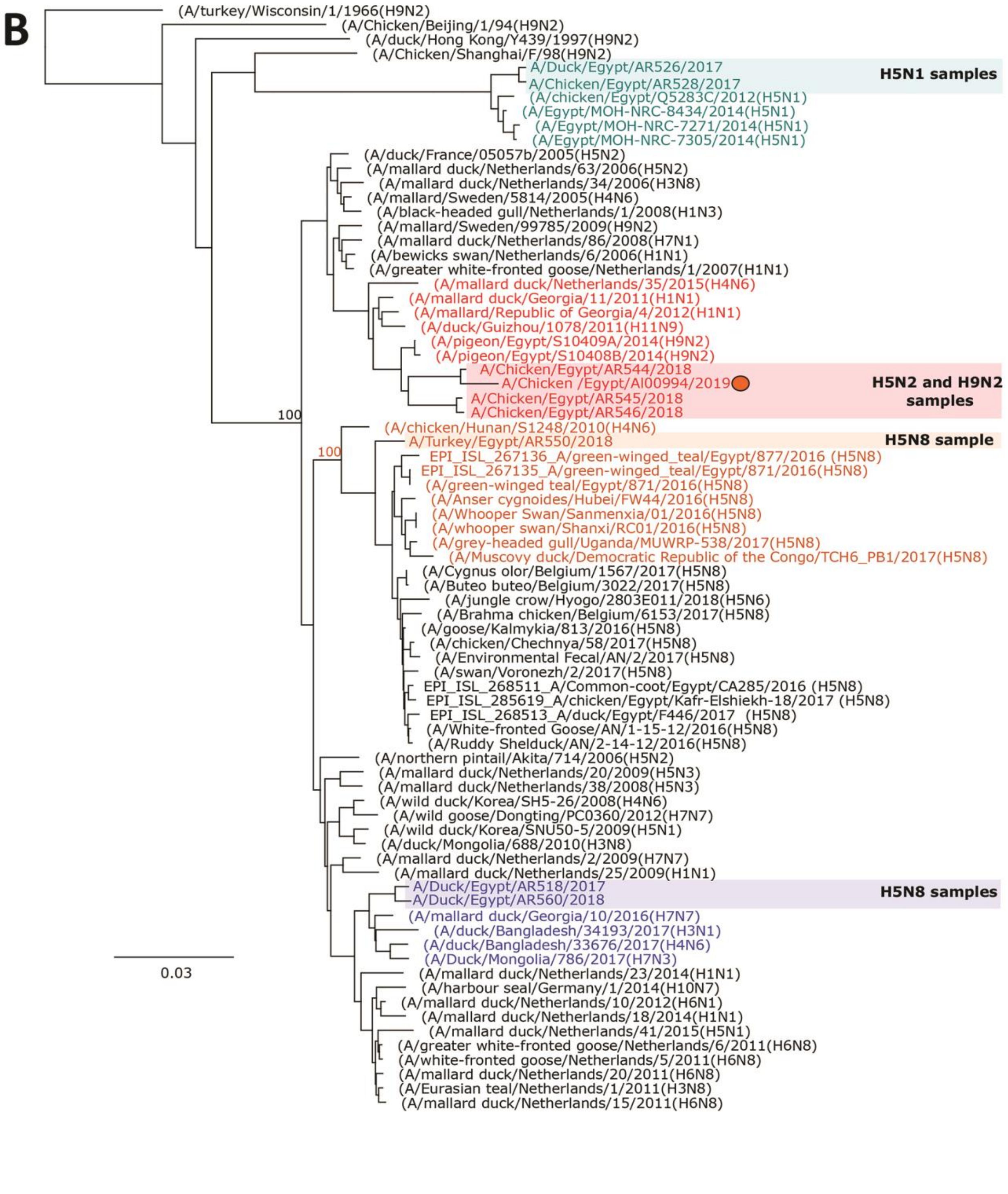
B
(A/turkey/Wisconsin/1/1966(H9N2)
(A/Chicken/Beijing/1/94(H9N2)
(A/duck/Hong Kong/Y439/1997(H9N2)
(A/Chicken/Shanghai/F/98(H9N2)
A/Duck/Egypt/AR526/2017
A/Chicken/Egypt/AR528/2017
H5N1 samples
(A/chicken/Egypt/Q5283C/2012(H5N1)
(A/Egypt/MOH-NRC-8434/2014(H5N1)
(A/Egypt/MOH-NRC-7271/2014(H5N1)
(A/Egypt/MOH-NRC-7305/2014(H5N1)
(A/duck/France/05057b/2005(H5N2)
(A/mallard duck/Netherlands/63/2006(H5N2)
(A/mallard duck/Netherlands/34/2006(H3N8)
(A/mallard/Sweden/5814/2005(H4N6)
(A/black-headed gull/Netherlands/1/2008(H1N3)
(A/mallard/Sweden/99785/2009(H9N2)
(A/mallard duck/Netherlands/86/2008(H7N1)
(A/bewicks swan/Netherlands/6/2006(H1N1)
(A/greater white-fronted goose/Netherlands/1/2007(H1N1)
(A/mallard duck/Netherlands/35/2015(H4N6)
(A/mallard duck/Georgia/11/2011(H1N1)
(A/mallard/Republic of Georgia/4/2012(H1N1)
(A/duck/Guizhou/1078/2011(H11N9)
(A/pigeon/Egypt/S10409A/2014(H9N2)
(A/pigeon/Egypt/S10408B/2014(H9N2)
A/Chicken/Egypt/AR544/2018
A/Chicken /Egypt/AI00994/2019
H5N2 and H9N2 samples
A/Chicken/Egypt/AR545/2018
A/Chicken/Egypt/AR546/2018
100
(A/chicken/Hunan/S1248/2010(H4N6)
A/Turkey/Egypt/AR550/2018
H5N8 sample
100
EPI_ISL_267136_A/green-winged_teal/Egypt/877/2016 (H5N8)
EPI_ISL_267135_A/green-winged_teal/Egypt/871/2016(H5N8)
(A/green-winged teal/Egypt/871/2016(H5N8)
(A/Anser cygnoides/Hubei/FW44/2016(H5N8)
(A/Whooper Swan/Sanmenxia/01/2016(H5N8)
(A/whooper swan/Shanxi/RC01/2016(H5N8)
(A/grey-headed gull/Uganda/MUWRP-538/2017(H5N8)
(A/Muscovy duck/Democratic Republic of the Congo/TCH6_PB1/2017(H5N8)
(A/Cygnus olor/Belgium/1567/2017(H5N8)
(A/Buteo buteo/Belgium/3022/2017(H5N8)
(A/jungle crow/Hyogo/2803E011/2018(H5N6)
(A/Brahma chicken/Belgium/6153/2017(H5N8)
(A/goose/Kalmykia/813/2016(H5N8)
(A/chicken/Chechnya/58/2017(H5N8)
(A/Environmental Fecal/AN/2/2017(H5N8)
(A/swan/Voronezh/2/2017(H5N8)
EPI_ISL_268511_A/Common-coot/Egypt/CA285/2016 (H5N8)
EPI_ISL_285619_A/chicken/Egypt/Kafr-Elshiekh-18/2017 (H5N8)
EPI_ISL_268513_A/duck/Egypt/F446/2017 (H5N8)
(A/White-fronted Goose/AN/1-15-12/2016(H5N8)
(A/Ruddy Shelduck/AN/2-14-12/2016(H5N8)
(A/northern pintail/Akita/714/2006(H5N2)
(A/mallard duck/Netherlands/20/2009(H5N3)
(A/mallard duck/Netherlands/38/2008(H5N3)
(A/wild duck/Korea/SH5-26/2008(H4N6)
(A/wild goose/Dongting/PC0360/2012(H7N7)
(A/wild duck/Korea/SNU50-5/2009(H5N1)
(A/duck/Mongolia/688/2010(H3N8)
(A/mallard duck/Netherlands/2/2009(H7N7)
(A/mallard duck/Netherlands/25/2009(H1N1)
A/Duck/Egypt/AR518/2017
A/Duck/Egypt/AR560/2018
H5N8 samples
(A/mallard duck/Georgia/10/2016(H7N7)
(A/duck/Bangladesh/34193/2017(H3N1)
(A/duck/Bangladesh/33676/2017(H4N6)
(A/Duck/Mongolia/786/2017(H7N3)
0.03
(A/mallard duck/Netherlands/23/2014(H1N1)
(A/harbour seal/Germany/1/2014(H10N7)
(A/mallard duck/Netherlands/10/2012(H6N1)
(A/mallard duck/Netherlands/18/2014(H1N1)
(A/mallard duck/Netherlands/41/2015(H5N1)
(A/greater white-fronted goose/Netherlands/6/2011(H6N8)
(A/white-fronted goose/Netherlands/5/2011(H6N8)
(A/mallard duck/Netherlands/20/2011(H6N8)
(A/Eurasian teal/Netherlands/1/2011(H3N8)
(A/mallard duck/Netherlands/15/2011(H6N8)

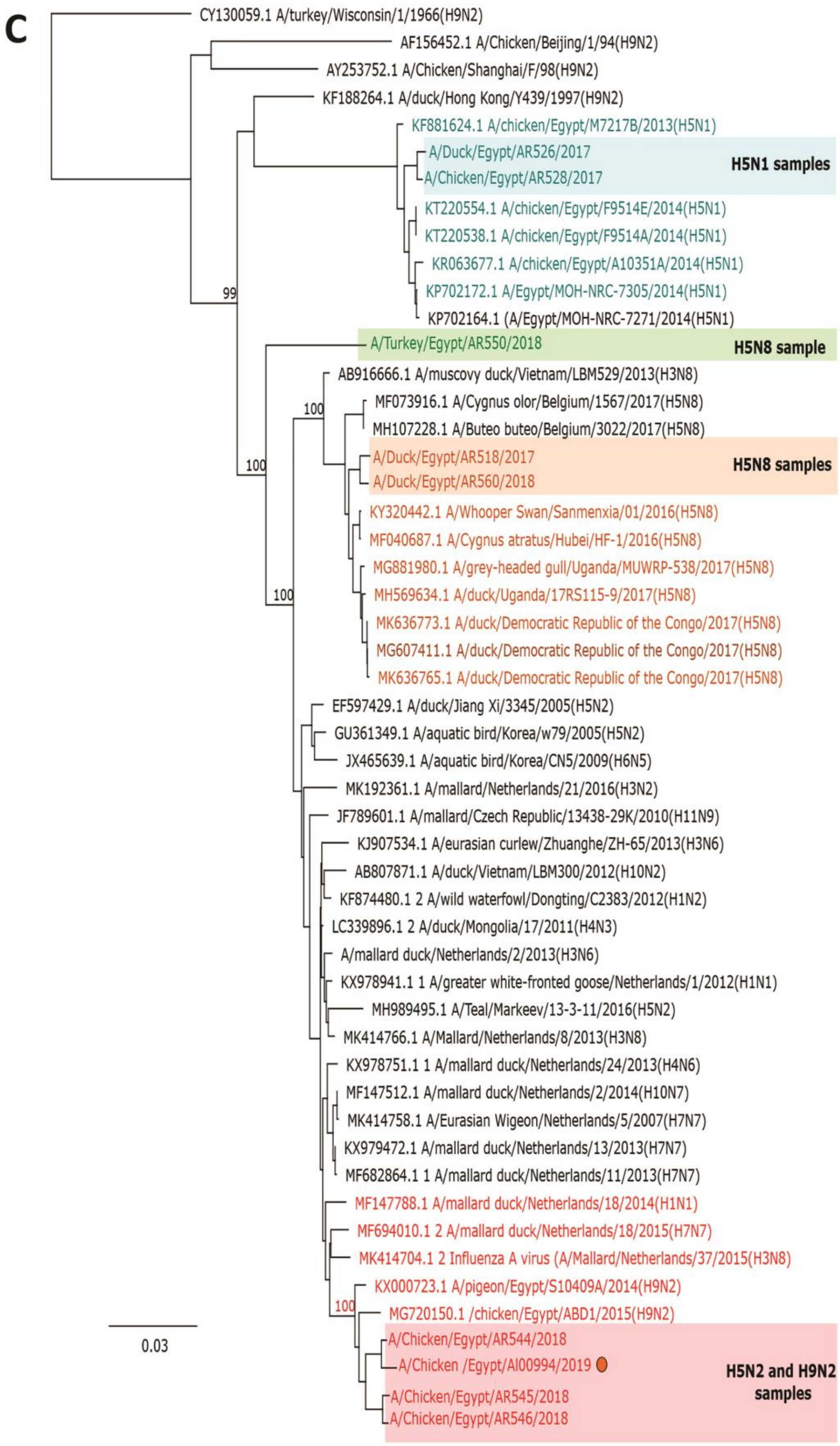
C
CY130059.1 A/turkey/Wisconsin/1/1966(H9N2)
AF156452.1 A/Chicken/Beijing/1/94(H9N2)
AY253752.1 A/Chicken/Shanghai/F/98(H9N2)
KF188264.1 A/duck/Hong Kong/Y439/1997(H9N2)
KF881624.1 A/chicken/Egypt/M7217B/2013(H5N1)
A/Duck/Egypt/AR526/2017
A/Chicken/Egypt/AR528/2017
H5N1 samples
KT220554.1 A/chicken/Egypt/F9514E/2014(H5N1)
KT220538.1 A/chicken/Egypt/F9514A/2014(H5N1)
KR063677.1 A/chicken/Egypt/A10351A/2014(H5N1)
KP702172.1 A/Egypt/MOH-NRC-7305/2014(H5N1)
KP702164.1 (A/Egypt/MOH-NRC-7271/2014(H5N1)
99
A/Turkey/Egypt/AR550/2018
H5N8 sample
AB916666.1 A/muscovy duck/Vietnam/LBM529/2013(H3N8)
100
MF073916.1 A/Cygnus olor/Belgium/1567/2017(H5N8)
MH107228.1 A/Buteo buteo/Belgium/3022/2017(H5N8)
100
A/Duck/Egypt/AR518/2017
A/Duck/Egypt/AR560/2018
H5N8 samples
KY320442.1 A/Whooper Swan/Sanmenxia/01/2016(H5N8)
MF040687.1 A/Cygnus atratus/Hubei/HF-1/2016(H5N8)
MG881980.1 A/grey-headed gull/Uganda/MUWRP-538/2017(H5N8)
100
MH569634.1 A/duck/Uganda/17RS115-9/2017(H5N8)
MK636773.1 A/duck/Democratic Republic of the Congo/2017(H5N8)
MG607411.1 A/duck/Democratic Republic of the Congo/2017(H5N8)
MK636765.1 A/duck/Democratic Republic of the Congo/2017(H5N8)
EF597429.1 A/duck/Jiang Xi/3345/2005(H5N2)
GU361349.1 A/aquatic bird/Korea/w79/2005(H5N2)
JX465639.1 A/aquatic bird/Korea/CN5/2009(H6N5)
MK192361.1 A/mallard/Netherlands/21/2016(H3N2)
JF789601.1 A/mallard/Czech Republic/13438-29K/2010(H11N9)
KJ907534.1 A/eurasian curlew/Zhuanghe/ZH-65/2013(H3N6)
AB807871.1 A/duck/Vietnam/LBM300/2012(H10N2)
KF874480.1 2 A/wild waterfowl/Dongting/C2383/2012(H1N2)
LC339896.1 2 A/duck/Mongolia/17/2011(H4N3)
A/mallard duck/Netherlands/2/2013(H3N6)
KX978941.1 1 A/greater white-fronted goose/Netherlands/1/2012(H1N1)
MH989495.1 A/Teal/Markeev/13-3-11/2016(H5N2)
MK414766.1 A/Mallard/Netherlands/8/2013(H3N8)
KX978751.1 1 A/mallard duck/Netherlands/24/2013(H4N6)
MF147512.1 A/mallard duck/Netherlands/2/2014(H10N7)
MK414758.1 A/Eurasian Wigeon/Netherlands/5/2007(H7N7)
KX979472.1 A/mallard duck/Netherlands/13/2013(H7N7)
MF682864.1 1 A/mallard duck/Netherlands/11/2013(H7N7)
MF147788.1 A/mallard duck/Netherlands/18/2014(H1N1)
MF694010.1 2 A/mallard duck/Netherlands/18/2015(H7N7)
MK414704.1 2 Influenza A virus (A/Mallard/Netherlands/37/2015(H3N8)
KX000723.1 A/pigeon/Egypt/S10409A/2014(H9N2)
100
MG720150.1 /chicken/Egypt/ABD1/2015(H9N2)
A/Chicken/Egypt/AR544/2018
A/Chicken /Egypt/AI00994/2019
A/Chicken/Egypt/AR545/2018
A/Chicken/Egypt/AR546/2018
H5N2 and H9N2 samples
0.03

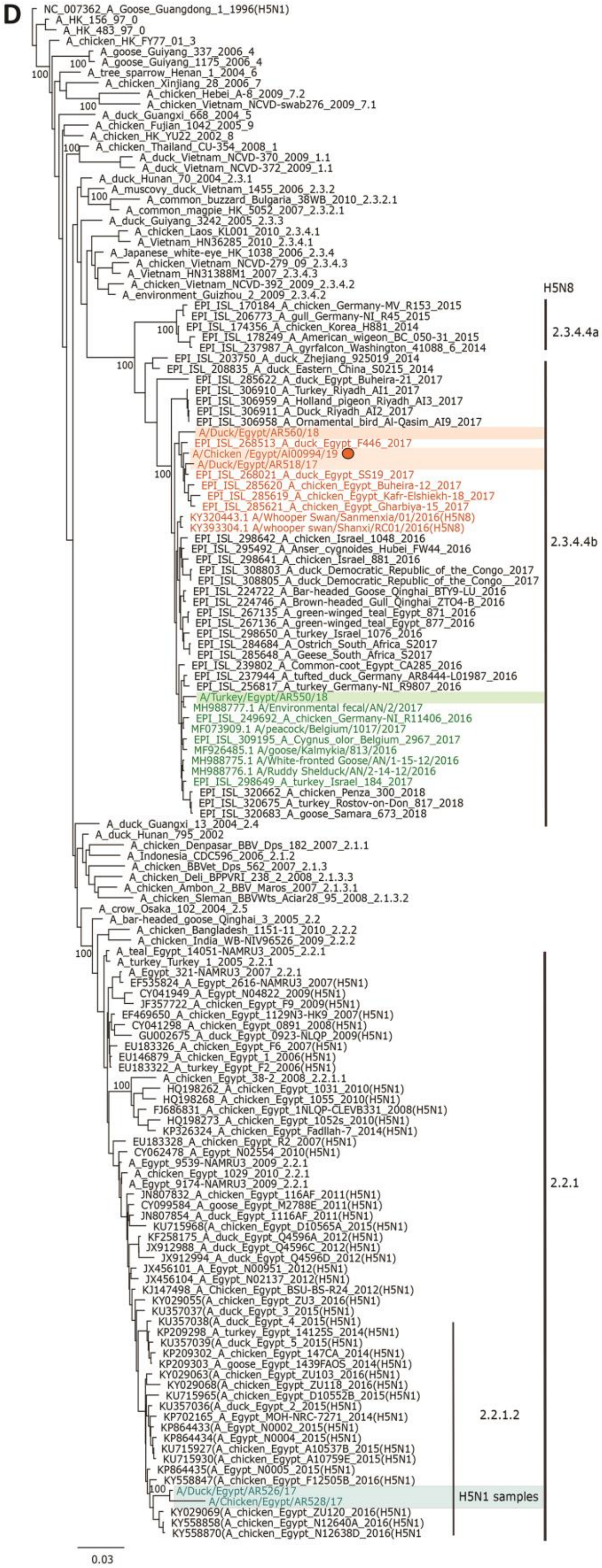
D
NC_007362_A_Goose_Guangdong_1_1996(H5N1)
A_HK_156_97_0
A_HK_483_97_0
A_chicken_HK_FY77_01_3
A_goose_Guiyang_337_2006_4
100
A_goose_Guiyang_1175_2006_4
100
A_tree_sparrow_Henan_1_2004_6
A_chicken_Xinjiang_28_2006_7
A_chicken_Hebei_A-8_2009_7.2
100
A_chicken_Vietnam_NCVD-swab276_2009_7.1
A_duck_Guangxi_668_2004_5
A_chicken_Fujian_1042_2005_9
A_chicken_HK_YU22_2002_8
100
A_chicken_Thailand_CU-354_2008_1
A_duck_Vietnam_NCVD-370_2009_1.1
A_duck_Vietnam_NCVD-372_2009_1.1
A_duck_Hunan_70_2004_2.3.1
A_muscovy_duck_Vietnam_1455_2006_2.3.2
100
A_common_buzzard_Bulgaria_38WB_2010_2.3.2.1
A_common_magpie_HK_5052_2007_2.3.2.1
A_duck_Guiyang_3242_2005_2.3.3
A_chicken_Laos_KL001_2010_2.3.4.1
A_Vietnam_HN36285_2010_2.3.4.1
A_Japanese_white-eye_HK_1038_2006_2.3.4
A_chicken_Vietnam_NCVD-279_09_2.3.4.3
A_Vietnam_HN31388M1_2007_2.3.4.3
A_chicken_Vietnam_NCVD-392_2009_2.3.4.2
A_environment_Guizhou_2_2009_2.3.4.2
H5N8
EPI_ISL_170184_A_chicken_Germany-MV_R153_2015
EPI_ISL_206773_A_gull_Germany-NI_R45_2015
100
EPI_ISL_174356_A_chicken_Korea_H881_2014
EPI_ISL_178249_A_American_wigeon_BC_050-31_2015
EPI_ISL_237987_A_gyrfalcon_Washington_41088_6_2014
2.3.4.4a
100
EPI_ISL_203750_A_duck_Zhejiang_925019_2014
EPI_ISL_208835_A_duck_Eastern_China_S0215_2014
EPI_ISL_285622_A_duck_Egypt_Buheira-21_2017
EPI_ISL_306910_A_Turkey_Riyadh_AI1_2017
EPI_ISL_306959_A_Holland_pigeon_Riyadh_AI3_2017
EPI_ISL_306911_A_Duck_Riyadh_AI2_2017
EPI_ISL_306958_A_Ornamental_bird_Al-Qasim_AI9_2017
A/Duck/Egypt/AR560/18
EPI_ISL_268513_A_duck_Egypt_F446_2017
A/Chicken /Egypt/AI00994/19
A/Duck/Egypt/AR518/17
100
EPI_ISL_268021_A_duck_Egypt_SS19_2017
EPI_ISL_285620_A_chicken_Egypt_Buheira-12_2017
EPI_ISL_285619_A_chicken_Egypt_Kafr-Elshiekh-18_2017
EPI_ISL_285621_A_chicken_Egypt_Gharbiya-15_2017
KY320443.1 A/Whooper Swan/Sanmenxia/01/2016(H5N8)
KY393304.1 A/whooper swan/Shanxi/RC01/2016(H5N8)
EPI_ISL_298642_A_chicken_Israel_1048_2016
EPI_ISL_295492_A_Anser_cygnoides_Hubei_FW44_2016
EPI_ISL_298641_A_chicken_Israel_881_2016
EPI_ISL_308803_A_duck_Democratic_Republic_of_the_Congo_2017
EPI_ISL_308805_A_duck_Democratic_Republic_of_the_Congo__2017
EPI_ISL_224722_A_Bar-headed_Goose_Qinghai_BTY9-LU_2016
EPI_ISL_224746_A_Brown-headed_Gull_Qinghai_ZTO4-B_2016
EPI_ISL_267135_A_green-winged_teal_Egypt_871_2016
EPI_ISL_267136_A_green-winged_teal_Egypt_877_2016
EPI_ISL_298650_A_turkey_Israel_1076_2016
EPI_ISL_284684_A_Ostrich_South_Africa_S2017
EPI_ISL_285648_A_Geese_South_Africa_S2017
EPI_ISL_239802_A_Common-coot_Egypt_CA285_2016
EPI_ISL_237944_A_tufted_duck_Germany_AR8444-L01987_2016
EPI_ISL_256817_A_turkey_Germany-NI_R9807_2016
A/Turkey/Egypt/AR550/18
MH988777.1 A/Environmental fecal/AN/2/2017
EPI_ISL_249692_A_chicken_Germany-NI_R11406_2016
MF073909.1 A/peacock/Belgium/1017/2017
EPI_ISL_309195_A_Cygnus_olor_Belgium_2967_2017
MF926485.1 A/goose/Kalmykia/813/2016
MH988775.1 A/White-fronted Goose/AN/1-15-12/2016
MH988776.1 A/Ruddy Shelduck/AN/2-14-12/2016
EPI_ISL_298649_A_turkey_Israel_184_2017
EPI_ISL_320662_A_chicken_Penza_300_2018
EPI_ISL_320675_A_turkey_Rostov-on-Don_817_2018
EPI_ISL_320683_A_goose_Samara_673_2018
2.3.4.4b
A_duck_Guangxi_13_2004_2.4
A_duck_Hunan_795_2002
A_chicken_Denpasar_BBV_Dps_182_2007_2.1.1
A_Indonesia_CDC596_2006_2.1.2
A_chicken_BBVet_Dps_562_2007_2.1.3
A_chicken_Deli_BPPVRI_238_2_2008_2.1.3.3
A_chicken_Ambon_2_BBV_Maros_2007_2.1.3.1
A_chicken_Sleman_BBVWts_Aciar28_95_2008_2.1.3.2
A_crow_Osaka_102_2004_2.5
A_bar-headed_goose_Qinghai_3_2005_2.2
A_chicken_Bangladesh_1151-11_2010_2.2.2
A_chicken_India_WB-NIV96526_2009_2.2.2
100
A_teal_Egypt_14051-NAMRU3_2005_2.2.1
A_turkey_Turkey_1_2005_2.2.1
A_Egypt_321-NAMRU3_2007_2.2.1
EF535824_A_Egypt_2616-NAMRU3_2007(H5N1)
CY041949_A_Egypt_N04822_2009(H5N1)
JF357722_A_chicken_Egypt_F9_2009(H5N1)
EF469650_A_chicken_Egypt_1129N3-HK9_2007(H5N1)
CY041298_A_chicken_Egypt_0891_2008(H5N1)
GU002675_A_duck_Egypt_0923-NLQP_2009(H5N1)
EU183326_A_chicken_Egypt_F6_2007(H5N1)
EU146879_A_chicken_Egypt_1_2006(H5N1)
EU183322_A_turkey_Egypt_F2_2006(H5N1)
A_chicken_Egypt_38-2_2008_2.2.1.1
100
HQ198262_A_chicken_Egypt_1031_2010(H5N1)
HQ198268_A_chicken_Egypt_1055_2010(H5N1)
FJ686831_A_chicken_Egypt_1NLQP-CLEVB331_2008(H5N1)
HQ198273_A_chicken_Egypt_1052s_2010(H5N1)
KP326324_A_chicken_Egypt_Fadllah-7_2014(H5N1)
EU183328_A_chicken_Egypt_R2_2007(H5N1)
CY062478_A_Egypt_N02554_2010(H5N1)
A_Egypt_9539-NAMRU3_2009_2.2.1
A_chicken_Egypt_1029_2010_2.2.1
A_Egypt_9174-NAMRU3_2009_2.2.1
JN807832_A_chicken_Egypt_116AF_2011(H5N1)
CY099584_A_goose_Egypt_M2788E_2011(H5N1)
JN807854_A_duck_Egypt_1116AF_2011(H5N1)
KU715968(A_chicken_Egypt_D10565A_2015(H5N1)
KF258175_A_duck_Egypt_Q4596A_2012(H5N1)
JX912988_A_duck_Egypt_Q4596C_2012(H5N1)
JX912994_A_duck_Egypt_Q4596D_2012(H5N1)
JX456101_A_Egypt_N00951_2012(H5N1)
JX456104_A_Egypt_N02137_2012(H5N1)
KJ147498_A_Chicken_Egypt_BSU-BS-R24_2012(H5N1)
KY029055(A_chicken_Egypt_ZU3_2016(H5N1)
KU357037(A_duck_Egypt_3_2015(H5N1)
2.2.1
KU357038(A_duck_Egypt_4_2015(H5N1)
KP209298_A_turkey_Egypt_14125S_2014(H5N1)
KU357039(A_duck_Egypt_5_2015(H5N1)
KP209302_A_chicken_Egypt_147CA_2014(H5N1)
KP209303_A_goose_Egypt_1439FAOS_2014(H5N1)
KY029063(A_chicken_Egypt_ZU103_2016(H5N1)
KY029068(A_chicken_Egypt_ZU118_2016(H5N1)
KU715965(A_chicken_Egypt_D10552B_2015(H5N1)
KU357036(A_duck_Egypt_2_2015(H5N1)
KP702165_A_Egypt_MOH-NRC-7271_2014(H5N1)
KP864433(A_Egypt_N0002_2015(H5N1)
KP864434(A_Egypt_N0004_2015(H5N1)
KU715927(A_chicken_Egypt_A10537B_2015(H5N1)
KU715930(A_chicken_Egypt_A10759E_2015(H5N1)
KP864435(A_Egypt_N0005_2015(H5N1)
KY558847(A_chicken_Egypt_F12505B_2016(H5N1)
2.2.1.2
100
A/Duck/Egypt/AR526/17
A/Chicken/Egypt/AR528/17
H5N1 samples
KY029069(A_chicken_Egypt_ZU120_2016(H5N1)
KY558858(A_chicken_Egypt_N12640A_2016(H5N1)
KY558870(A_chicken_Egypt_N12638D_2016(H5N1
0.03

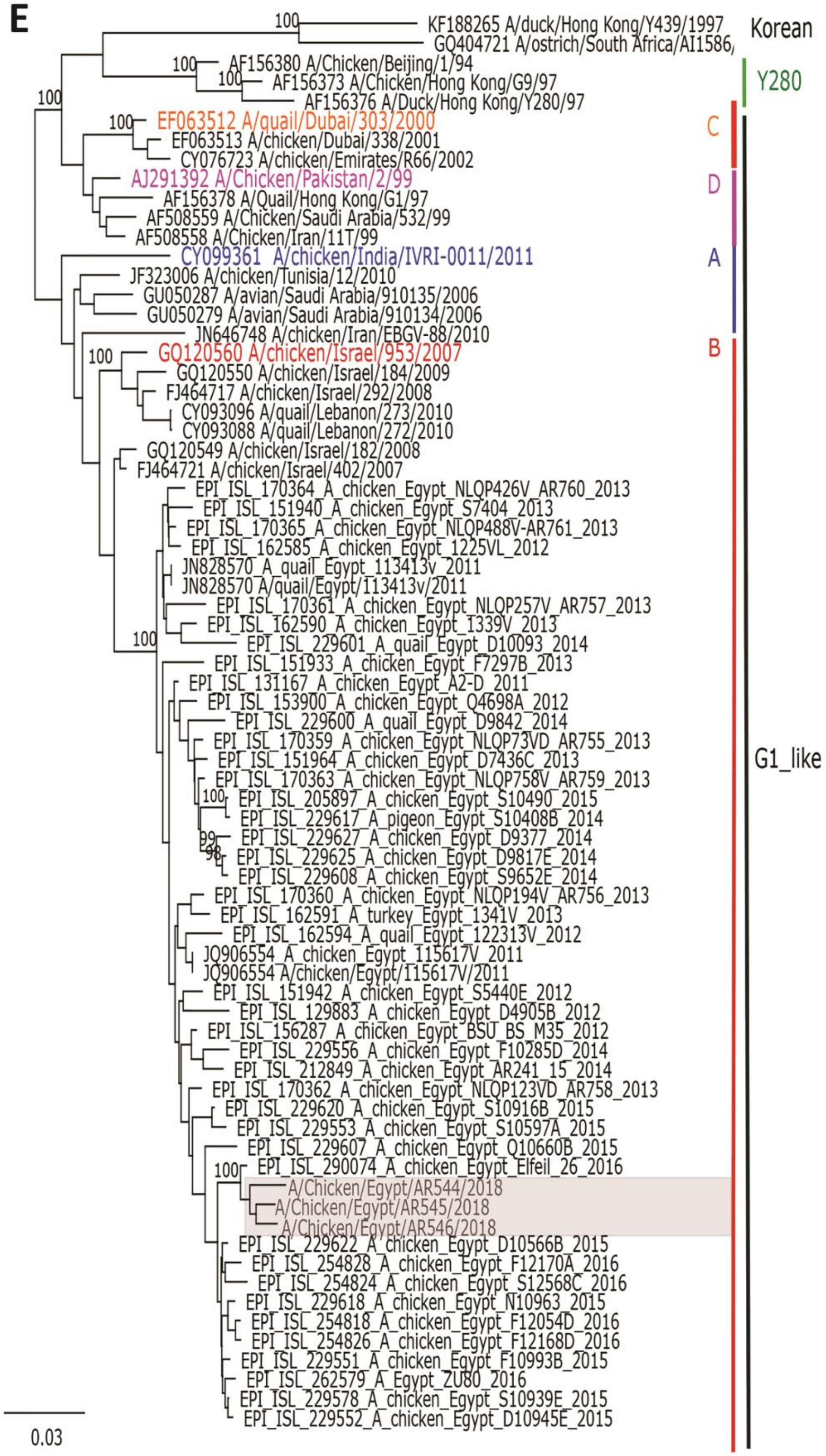
E
KF188265 A/duck/Hong Kong/Y439/1997
GQ404721 A/ostrich/South Africa/AI1586/
Korean
AF156380 A/Chicken/Beijing/1/94
AF156373 A/Chicken/Hong Kong/G9/97
AF156376 A/Duck/Hong Kong/Y280/97
Y280
EF063512 A/quail/Dubai/303/2000
EF063513 A/chicken/Dubai/338/2001
CY076723 A/chicken/Emirates/R66/2002
C
AJ291392 A/Chicken/Pakistan/2/99
AF156378 A/Quail/Hong Kong/G1/97
AF508559 A/Chicken/Saudi Arabia/532/99
AF508558 A/Chicken/Iran/11T/99
D
CY099361 A/chicken/India/IVRI-0011/2011
JF323006 A/chicken/Tunisia/12/2010
GU050287 A/avian/Saudi Arabia/910135/2006
GU050279 A/avian/Saudi Arabia/910134/2006
A
JN646748 A/chicken/Iran/EBGV-88/2010
GQ120560 A/chicken/Israel/953/2007
GQ120550 A/chicken/Israel/184/2009
FJ464717 A/chicken/Israel/292/2008
CY093096 A/quail/Lebanon/273/2010
CY093088 A/quail/Lebanon/272/2010
GQ120549 A/chicken/Israel/182/2008
FJ464721 A/chicken/Israel/402/2007
B
EPI_ISL_170364_A_chicken_Egypt_NLQP426V_AR760_2013
EPI_ISL_151940_A_chicken_Egypt_S7404_2013
EPI_ISL_170365_A_chicken_Egypt_NLQP488V-AR761_2013
EPI_ISL_162585_A_chicken_Egypt_1225VL_2012
JN828570_A_quail_Egypt_113413v_2011
JN828570 A/quail/Egypt/113413v/2011
EPI_ISL_170361_A_chicken_Egypt_NLQP257V_AR757_2013
EPI_ISL_162590_A_chicken_Egypt_1339V_2013
EPI_ISL_229601_A_quail_Egypt_D10093_2014
EPI_ISL_151933_A_chicken_Egypt_F7297B_2013
EPI_ISL_131167_A_chicken_Egypt_A2-D_2011
EPI_ISL_153900_A_chicken_Egypt_Q4698A_2012
EPI_ISL_229600_A_quail_Egypt_D9842_2014
EPI_ISL_170359_A_chicken_Egypt_NLQP73VD_AR755_2013
EPI_ISL_151964_A_chicken_Egypt_D7436C_2013
EPI_ISL_170363_A_chicken_Egypt_NLQP758V_AR759_2013
EPI_ISL_205897_A_chicken_Egypt_S10490_2015
EPI_ISL_229617_A_pigeon_Egypt_S10408B_2014
EPI_ISL_229627_A_chicken_Egypt_D9377_2014
EPI_ISL_229625_A_chicken_Egypt_D9817E_2014
EPI_ISL_229608_A_chicken_Egypt_S9652E_2014
EPI_ISL_170360_A_chicken_Egypt_NLQP194V_AR756_2013
EPI_ISL_162591_A_turkey_Egypt_1341V_2013
EPI_ISL_162594_A_quail_Egypt_122313V_2012
JQ906554_A_chicken_Egypt_115617V_2011
JQ906554 A/chicken/Egypt/115617V/2011
EPI_ISL_151942_A_chicken_Egypt_S5440E_2012
EPI_ISL_129883_A_chicken_Egypt_D4905B_2012
EPI_ISL_156287_A_chicken_Egypt_BSU_BS_M35_2012
EPI_ISL_229556_A_chicken_Egypt_F10285D_2014
EPI_ISL_212849_A_chicken_Egypt_AR241_15_2014
EPI_ISL_170362_A_chicken_Egypt_NLQP123VD_AR758_2013
EPI_ISL_229620_A_chicken_Egypt_S10916B_2015
EPI_ISL_229553_A_chicken_Egypt_S10597A_2015
EPI_ISL_229607_A_chicken_Egypt_Q10660B_2015
EPI_ISL_290074_A_chicken_Egypt_Elfeil_26_2016
A/Chicken/Egypt/AR544/2018
A/Chicken/Egypt/AR545/2018
A/Chicken/Egypt/AR546/2018
EPI_ISL_229622_A_chicken_Egypt_D10566B_2015
EPI_ISL_254828_A_chicken_Egypt_F12170A_2016
EPI_ISL_254824_A_chicken_Egypt_S12568C_2016
EPI_ISL_229618_A_chicken_Egypt_N10963_2015
EPI_ISL_254818_A_chicken_Egypt_F12054D_2016
EPI_ISL_254826_A_chicken_Egypt_F12168D_2016
EPI_ISL_229551_A_chicken_Egypt_F10993B_2015
EPI_ISL_262579_A_Egypt_ZU80_2016
EPI_ISL_229578_A_chicken_Egypt_S10939E_2015
EPI_ISL_229552_A_chicken_Egypt_D10945E_2015
G1_like
0.03

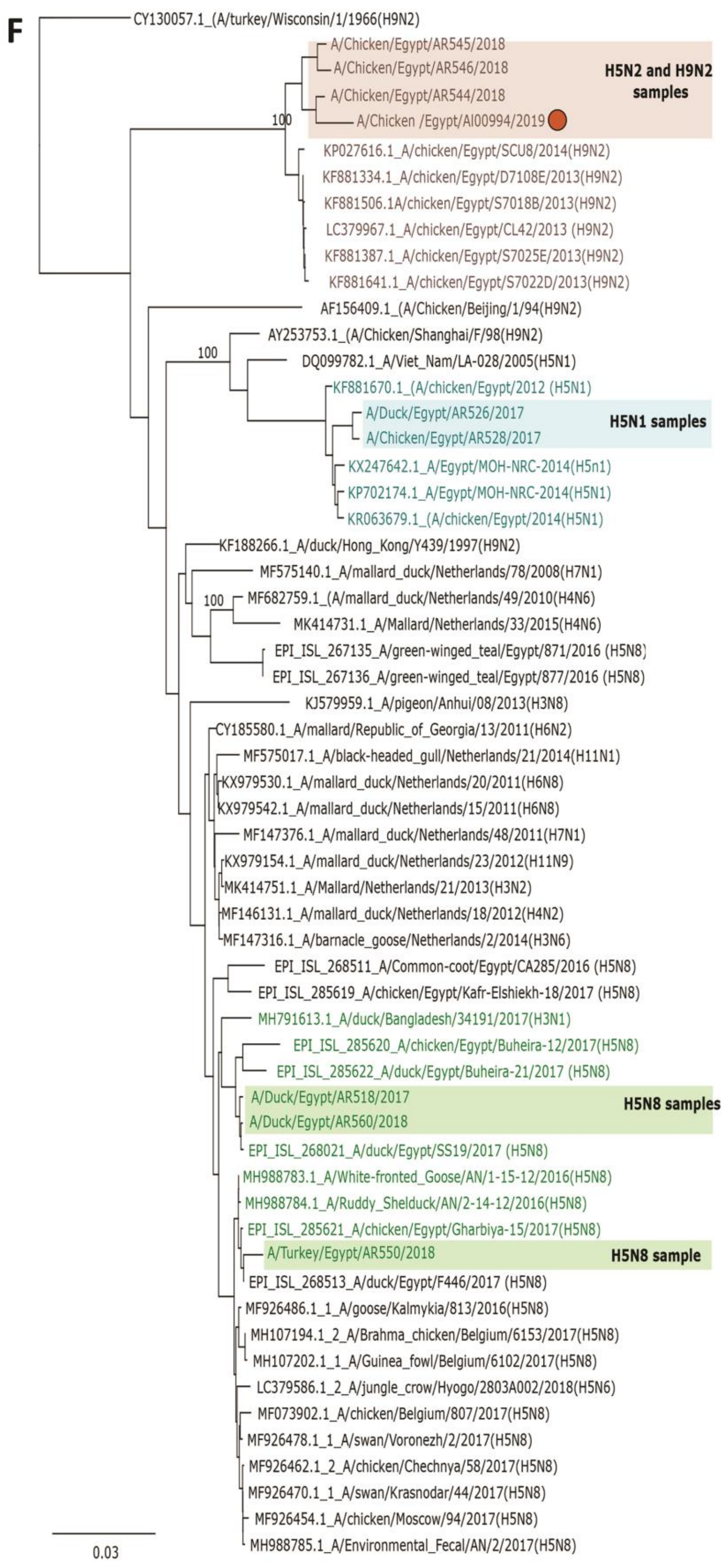
F
CY130057.1_(A/turkey/Wisconsin/1/1966(H9N2)
A/Chicken/Egypt/AR545/2018
A/Chicken/Egypt/AR546/2018
A/Chicken/Egypt/AR544/2018
A/Chicken /Egypt/AI00994/2019
H5N2 and H9N2 samples
100
KP027616.1_A/chicken/Egypt/SCU8/2014(H9N2)
KF881334.1_A/chicken/Egypt/D7108E/2013(H9N2)
KF881506.1A/chicken/Egypt/S7018B/2013(H9N2)
LC379967.1_A/chicken/Egypt/CL42/2013 (H9N2)
KF881387.1_A/chicken/Egypt/S7025E/2013(H9N2)
KF881641.1_A/chicken/Egypt/S7022D/2013(H9N2)
AF156409.1_(A/Chicken/Beijing/1/94(H9N2)
AY253753.1_(A/Chicken/Shanghai/F/98(H9N2)
100
DQ099782.1_A/Viet_Nam/LA-028/2005(H5N1)
KF881670.1_(A/chicken/Egypt/2012 (H5N1)
A/Duck/Egypt/AR526/2017
A/Chicken/Egypt/AR528/2017
H5N1 samples
KX247642.1_A/Egypt/MOH-NRC-2014(H5n1)
KP702174.1_A/Egypt/MOH-NRC-2014(H5N1)
KR063679.1_(A/chicken/Egypt/2014(H5N1)
KF188266.1_A/duck/Hong_Kong/Y439/1997(H9N2)
MF575140.1_A/mallard_duck/Netherlands/78/2008(H7N1)
100
MF682759.1_(A/mallard_duck/Netherlands/49/2010(H4N6)
MK414731.1_A/Mallard/Netherlands/33/2015(H4N6)
EPI_ISL_267135_A/green-winged_teal/Egypt/871/2016 (H5N8)
EPI_ISL_267136_A/green-winged_teal/Egypt/877/2016 (H5N8)
KJ579959.1_A/pigeon/Anhui/08/2013(H3N8)
CY185580.1_A/mallard/Republic_of_Georgia/13/2011(H6N2)
MF575017.1_A/black-headed_gull/Netherlands/21/2014(H11N1)
KX979530.1_A/mallard_duck/Netherlands/20/2011(H6N8)
KX979542.1_A/mallard_duck/Netherlands/15/2011(H6N8)
MF147376.1_A/mallard_duck/Netherlands/48/2011(H7N1)
KX979154.1_A/mallard_duck/Netherlands/23/2012(H11N9)
MK414751.1_A/Mallard/Netherlands/21/2013(H3N2)
MF146131.1_A/mallard_duck/Netherlands/18/2012(H4N2)
MF147316.1_A/barnacle_goose/Netherlands/2/2014(H3N6)
EPI_ISL_268511_A/Common-coot/Egypt/CA285/2016 (H5N8)
EPI_ISL_285619_A/chicken/Egypt/Kafr-Elshiekh-18/2017 (H5N8)
MH791613.1_A/duck/Bangladesh/34191/2017(H3N1)
EPI_ISL_285620_A/chicken/Egypt/Buheira-12/2017(H5N8)
EPI_ISL_285622_A/duck/Egypt/Buheira-21/2017 (H5N8)
A/Duck/Egypt/AR518/2017
A/Duck/Egypt/AR560/2018
H5N8 samples
EPI_ISL_268021_A/duck/Egypt/SS19/2017 (H5N8)
MH988783.1_A/White-fronted_Goose/AN/1-15-12/2016(H5N8)
MH988784.1_A/Ruddy_Shelduck/AN/2-14-12/2016(H5N8)
EPI_ISL_285621_A/chicken/Egypt/Gharbiya-15/2017(H5N8)
A/Turkey/Egypt/AR550/2018
H5N8 sample
EPI_ISL_268513_A/duck/Egypt/F446/2017 (H5N8)
MF926486.1_1_A/goose/Kalmykia/813/2016(H5N8)
MH107194.1_2_A/Brahma_chicken/Belgium/6153/2017(H5N8)
MH107202.1_1_A/Guinea_fowl/Belgium/6102/2017(H5N8)
LC379586.1_2_A/jungle_crow/Hyogo/2803A002/2018(H5N6)
MF073902.1_A/chicken/Belgium/807/2017(H5N8)
MF926478.1_1_A/swan/Voronezh/2/2017(H5N8)
MF926462.1_2_A/chicken/Chechnya/58/2017(H5N8)
MF926470.1_1_A/swan/Krasnodar/44/2017(H5N8)
MF926454.1_A/chicken/Moscow/94/2017(H5N8)
MH988785.1_A/Environmental_Fecal/AN/2/2017(H5N8)
0.03

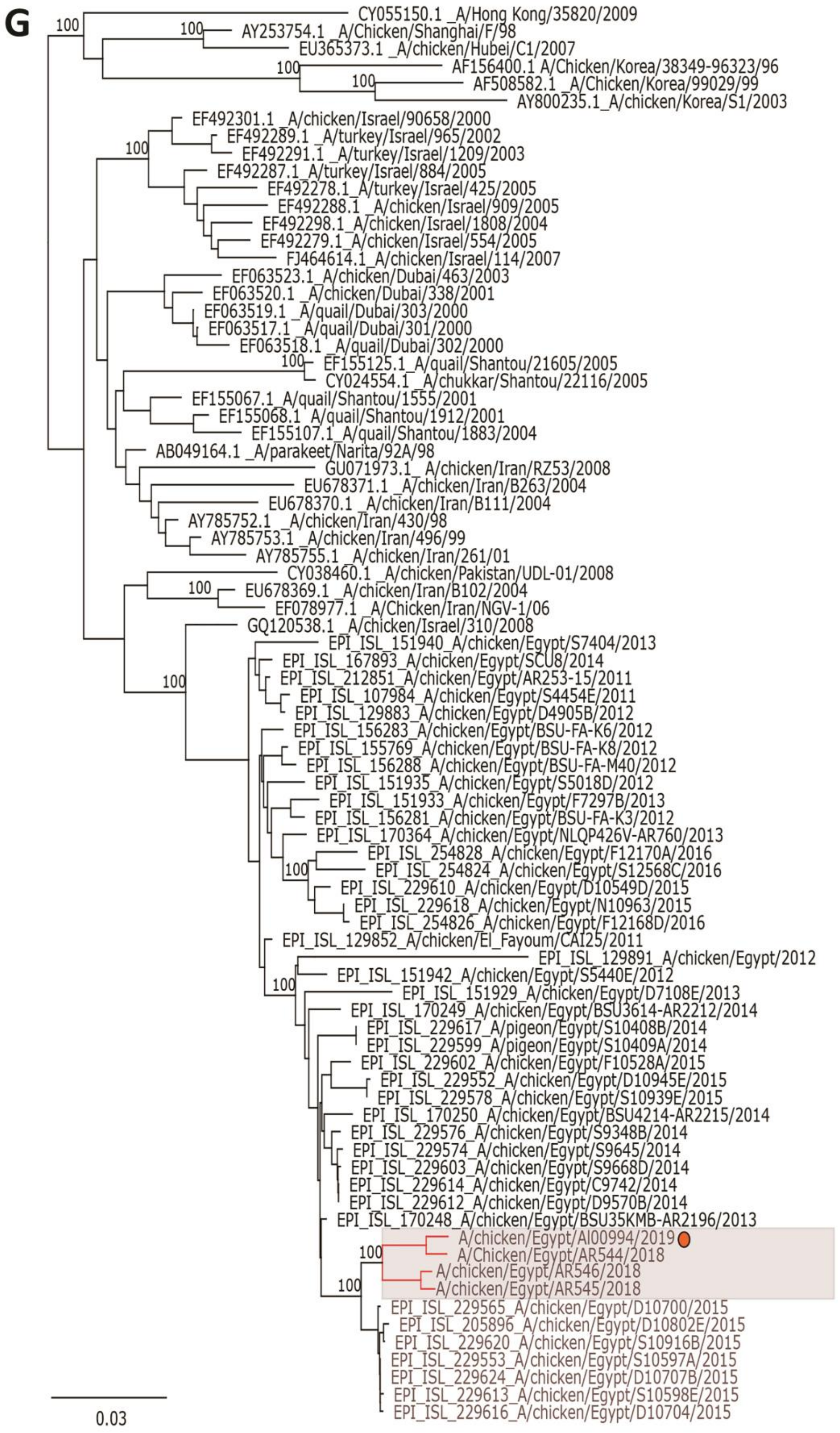
G
CY055150.1 _A/Hong Kong/35820/2009
AY253754.1 _A/Chicken/Shanghai/F/98
EU365373.1 _A/chicken/Hubei/C1/2007
AF156400.1 A/Chicken/Korea/38349-96323/96
AF508582.1 _A/Chicken/Korea/99029/99
AY800235.1_A/chicken/Korea/S1/2003
EF492301.1 _A/chicken/Israel/90658/2000
EF492289.1 _A/turkey/Israel/965/2002
EF492291.1 _A/turkey/Israel/1209/2003
EF492287.1_A/turkey/Israel/884/2005
EF492278.1_A/turkey/Israel/425/2005
EF492288.1 _A/chicken/Israel/909/2005
EF492298.1_A/chicken/Israel/1808/2004
EF492279.1_A/chicken/Israel/554/2005
FJ464614.1_A/chicken/Israel/114/2007
EF063523.1_A/chicken/Dubai/463/2003
EF063520.1 _A/chicken/Dubai/338/2001
EF063519.1 _A/quail/Dubai/303/2000
EF063517.1 _A/quail/Dubai/301/2000
EF063518.1 _A/quail/Dubai/302/2000
EF155125.1_A/quail/Shantou/21605/2005
CY024554.1 _A/chukkar/Shantou/22116/2005
EF155067.1_A/quail/Shantou/1555/2001
EF155068.1_A/quail/Shantou/1912/2001
EF155107.1_A/quail/Shantou/1883/2004
AB049164.1 _A/parakeet/Narita/92A/98
GU071973.1_ A/chicken/Iran/RZ53/2008
EU678371.1 _A/chicken/Iran/B263/2004
EU678370.1 _A/chicken/Iran/B111/2004
AY785752.1 _A/chicken/Iran/430/98
AY785753.1 _A/chicken/Iran/496/99
AY785755.1 _A/chicken/Iran/261/01
CY038460.1 _A/chicken/Pakistan/UDL-01/2008
EU678369.1 _A/chicken/Iran/B102/2004
EF078977.1 _A/Chicken/Iran/NGV-1/06
GQ120538.1 _A/chicken/Israel/310/2008
EPI_ISL_151940_A/chicken/Egypt/S7404/2013
EPI_ISL_167893_A/chicken/Egypt/SCU8/2014
EPI_ISL_212851_A/chicken/Egypt/AR253-15/2011
EPI_ISL_107984_A/chicken/Egypt/S4454E/2011
EPI_ISL_129883_A/chicken/Egypt/D4905B/2012
EPI_ISL_156283_A/chicken/Egypt/BSU-FA-K6/2012
EPI_ISL_155769_A/chicken/Egypt/BSU-FA-K8/2012
EPI_ISL_156288_A/chicken/Egypt/BSU-FA-M40/2012
EPI_ISL_151935_A/chicken/Egypt/S5018D/2012
EPI_ISL_151933_A/chicken/Egypt/F7297B/2013
EPI_ISL_156281_A/chicken/Egypt/BSU-FA-K3/2012
EPI_ISL_170364_A/chicken/Egypt/NLQP426V-AR760/2013
EPI_ISL_254828_A/chicken/Egypt/F12170A/2016
EPI_ISL_254824_A/chicken/Egypt/S12568C/2016
EPI_ISL_229610_A/chicken/Egypt/D10549D/2015
EPI_ISL_229618_A/chicken/Egypt/N10963/2015
EPI_ISL_254826_A/chicken/Egypt/F12168D/2016
EPI_ISL_129852_A/chicken/El_Fayoum/CAI25/2011
EPI_ISL_129891_A/chicken/Egypt/2012
EPI_ISL_151942_A/chicken/Egypt/S5440E/2012
EPI_ISL_151929_A/chicken/Egypt/D7108E/2013
EPI_ISL_170249_A/chicken/Egypt/BSU3614-AR2212/2014
EPI_ISL_229617_A/pigeon/Egypt/S10408B/2014
EPI_ISL_229599_A/pigeon/Egypt/S10409A/2014
EPI_ISL_229602_A/chicken/Egypt/F10528A/2015
EPI_ISL_229552_A/chicken/Egypt/D10945E/2015
EPI_ISL_229578_A/chicken/Egypt/S10939E/2015
EPI_ISL_170250_A/chicken/Egypt/BSU4214-AR2215/2014
EPI_ISL_229576_A/chicken/Egypt/S9348B/2014
EPI_ISL_229574_A/chicken/Egypt/S9645/2014
EPI_ISL_229603_A/chicken/Egypt/S9668D/2014
EPI_ISL_229614_A/chicken/Egypt/C9742/2014
EPI_ISL_229612_A/chicken/Egypt/D9570B/2014
EPI_ISL_170248_A/chicken/Egypt/BSU35KMB-AR2196/2013
A/chicken/Egypt/AI00994/2019
A/Chicken/Egypt/AR544/2018
A/chicken/Egypt/AR546/2018
A/chicken/Egypt/AR545/2018
EPI_ISL_229565_A/chicken/Egypt/D10700/2015
EPI_ISL_205896_A/chicken/Egypt/D10802E/2015
EPI_ISL_229620_A/chicken/Egypt/S10916B/2015
EPI_ISL_229553_A/chicken/Egypt/S10597A/2015
EPI_ISL_229624_A/chicken/Egypt/D10707B/2015
EPI_ISL_229613_A/chicken/Egypt/S10598E/2015
EPI_ISL_229616_A/chicken/Egypt/D10704/2015
0.03

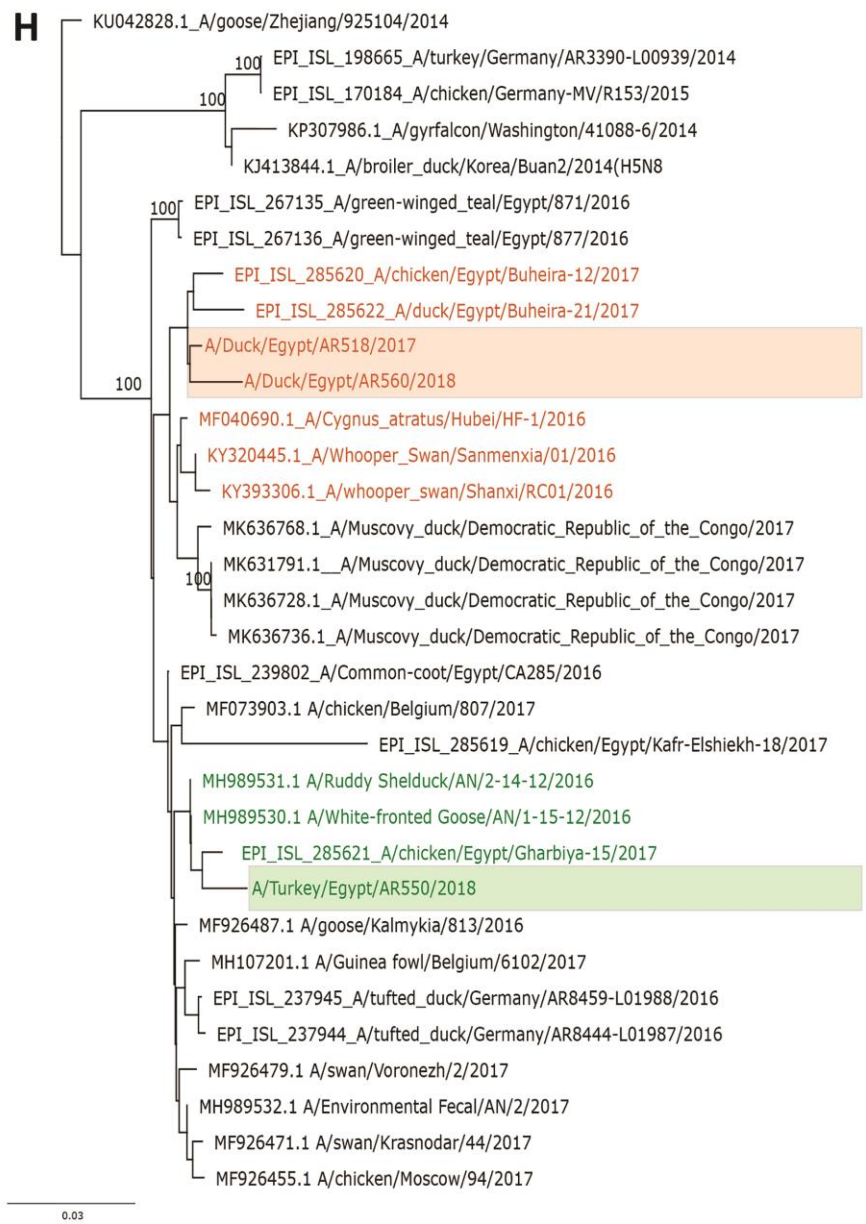
H
KU042828.1_A/goose/Zhejiang/925104/2014
EPI_ISL_198665_A/turkey/Germany/AR3390-L00939/2014
EPI_ISL_170184_A/chicken/Germany-MV/R153/2015
KP307986.1_A/gyrfalcon/Washington/41088-6/2014
KJ413844.1_A/broiler_duck/Korea/Buan2/2014(H5N8
EPI_ISL_267135_A/green-winged_teal/Egypt/871/2016
EPI_ISL_267136_A/green-winged_teal/Egypt/877/2016
EPI_ISL_285620_A/chicken/Egypt/Buheira-12/2017
EPI_ISL_285622_A/duck/Egypt/Buheira-21/2017
A/Duck/Egypt/AR518/2017
A/Duck/Egypt/AR560/2018
MF040690.1_A/Cygnus_atratus/Hubei/HF-1/2016
KY320445.1_A/Whooper_Swan/Sanmenxia/01/2016
KY393306.1_A/whooper_swan/Shanxi/RC01/2016
MK636768.1_A/Muscovy_duck/Democratic_Republic_of_the_Congo/2017
MK631791.1__A/Muscovy_duck/Democratic_Republic_of_the_Congo/2017
MK636728.1_A/Muscovy_duck/Democratic_Republic_of_the_Congo/2017
MK636736.1_A/Muscovy_duck/Democratic_Republic_of_the_Congo/2017
EPI_ISL_239802_A/Common-coot/Egypt/CA285/2016
MF073903.1 A/chicken/Belgium/807/2017
EPI_ISL_285619_A/chicken/Egypt/Kafr-Elshiekh-18/2017
MH989531.1 A/Ruddy Shelduck/AN/2-14-12/2016
MH989530.1 A/White-fronted Goose/AN/1-15-12/2016
EPI_ISL_285621_A/chicken/Egypt/Gharbiya-15/2017
A/Turkey/Egypt/AR550/2018
MF926487.1 A/goose/Kalmykia/813/2016
MH107201.1 A/Guinea fowl/Belgium/6102/2017
EPI_ISL_237945_A/tufted_duck/Germany/AR8459-L01988/2016
EPI_ISL_237944_A/tufted_duck/Germany/AR8444-L01987/2016
MF926479.1 A/swan/Voronezh/2/2017
MH989532.1 A/Environmental Fecal/AN/2/2017
MF926471.1 A/swan/Krasnodar/44/2017
MF926455.1 A/chicken/Moscow/94/2017
100
100
100
100
100
0.03

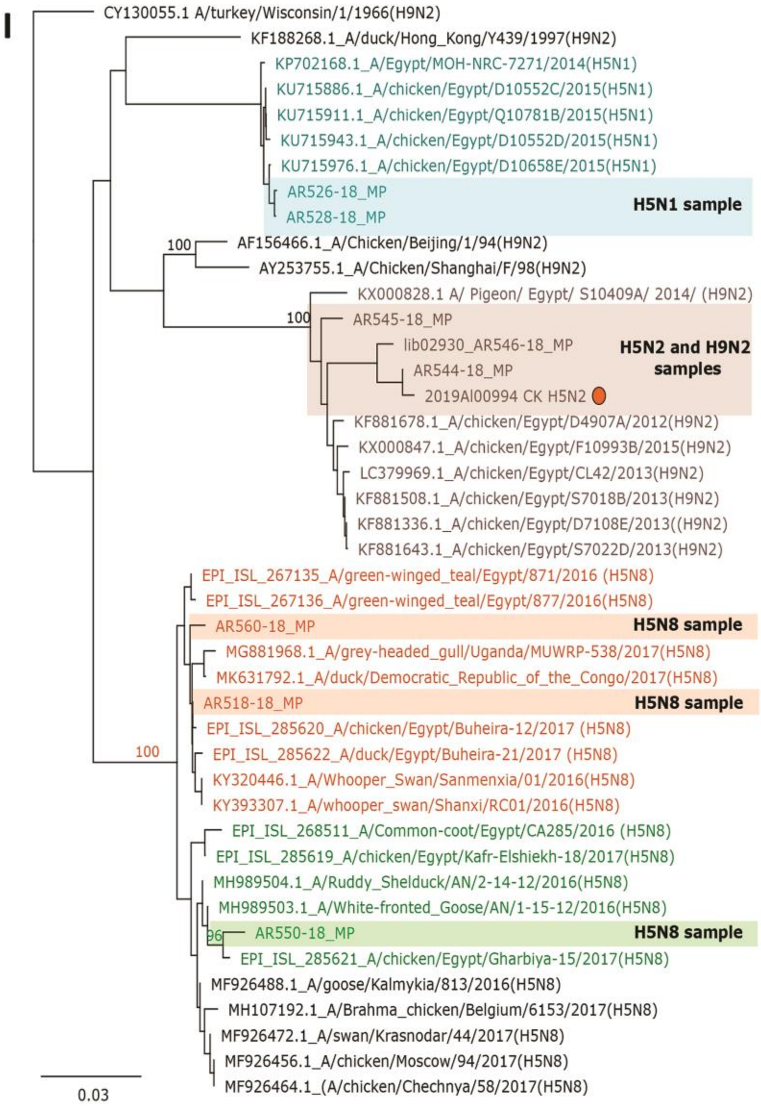

CY130055.1 A/turkey/Wisconsin/1/1966(H9N2)
KF188268.1_A/duck/Hong_Kong/Y439/1997(H9N2)
KP702168.1_A/Egypt/MOH-NRC-7271/2014(H5N1)
KU715886.1_A/chicken/Egypt/D10552C/2015(H5N1)
KU715911.1_A/chicken/Egypt/Q10781B/2015(H5N1)
KU715943.1_A/chicken/Egypt/D10552D/2015(H5N1)
KU715976.1_A/chicken/Egypt/D10658E/2015(H5N1)
AR526-18_MP
AR528-18_MP
H5N1 sample
100
AF156466.1_A/Chicken/Beijing/1/94(H9N2)
AY253755.1_A/Chicken/Shanghai/F/98(H9N2)
KX000828.1 A/ Pigeon/ Egypt/ S10409A/ 2014/ (H9N2)
100
AR545-18_MP
lib02930_AR546-18_MP
AR544-18_MP
2019AI00994 CK H5N2
H5N2 and H9N2 samples
KF881678.1_A/chicken/Egypt/D4907A/2012(H9N2)
KX000847.1_A/chicken/Egypt/F10993B/2015(H9N2)
LC379969.1_A/chicken/Egypt/CL42/2013(H9N2)
KF881508.1_A/chicken/Egypt/S7018B/2013(H9N2)
KF881336.1_A/chicken/Egypt/D7108E/2013((H9N2)
KF881643.1_A/chicken/Egypt/S7022D/2013(H9N2)
EPI_ISL_267135_A/green-winged_teal/Egypt/871/2016 (H5N8)
EPI_ISL_267136_A/green-winged_teal/Egypt/877/2016(H5N8)
AR560-18_MP
H5N8 sample
MG881968.1_A/grey-headed_gull/Uganda/MUWRP-538/2017(H5N8)
MK631792.1_A/duck/Democratic_Republic_of_the_Congo/2017(H5N8)
AR518-18_MP
H5N8 sample
EPI_ISL_285620_A/chicken/Egypt/Buheira-12/2017 (H5N8)
100
EPI_ISL_285622_A/duck/Egypt/Buheira-21/2017 (H5N8)
KY320446.1_A/Whooper_Swan/Sanmenxia/01/2016(H5N8)
KY393307.1_A/whooper_swan/Shanxi/RC01/2016(H5N8)
EPI_ISL_268511_A/Common-coot/Egypt/CA285/2016 (H5N8)
EPI_ISL_285619_A/chicken/Egypt/Kafr-Elshiekh-18/2017(H5N8)
MH989504.1_A/Ruddy_Shelduck/AN/2-14-12/2016(H5N8)
MH989503.1_A/White-fronted_Goose/AN/1-15-12/2016(H5N8)
96
AR550-18_MP
H5N8 sample
EPI_ISL_285621_A/chicken/Egypt/Gharbiya-15/2017(H5N8)
MF926488.1_A/goose/Kalmykia/813/2016(H5N8)
MH107192.1_A/Brahma_chicken/Belgium/6153/2017(H5N8)
MF926472.1_A/swan/Krasnodar/44/2017(H5N8)
MF926456.1_A/chicken/Moscow/94/2017(H5N8)
MF926464.1_(A/chicken/Chechnya/58/2017(H5N8)
0.03

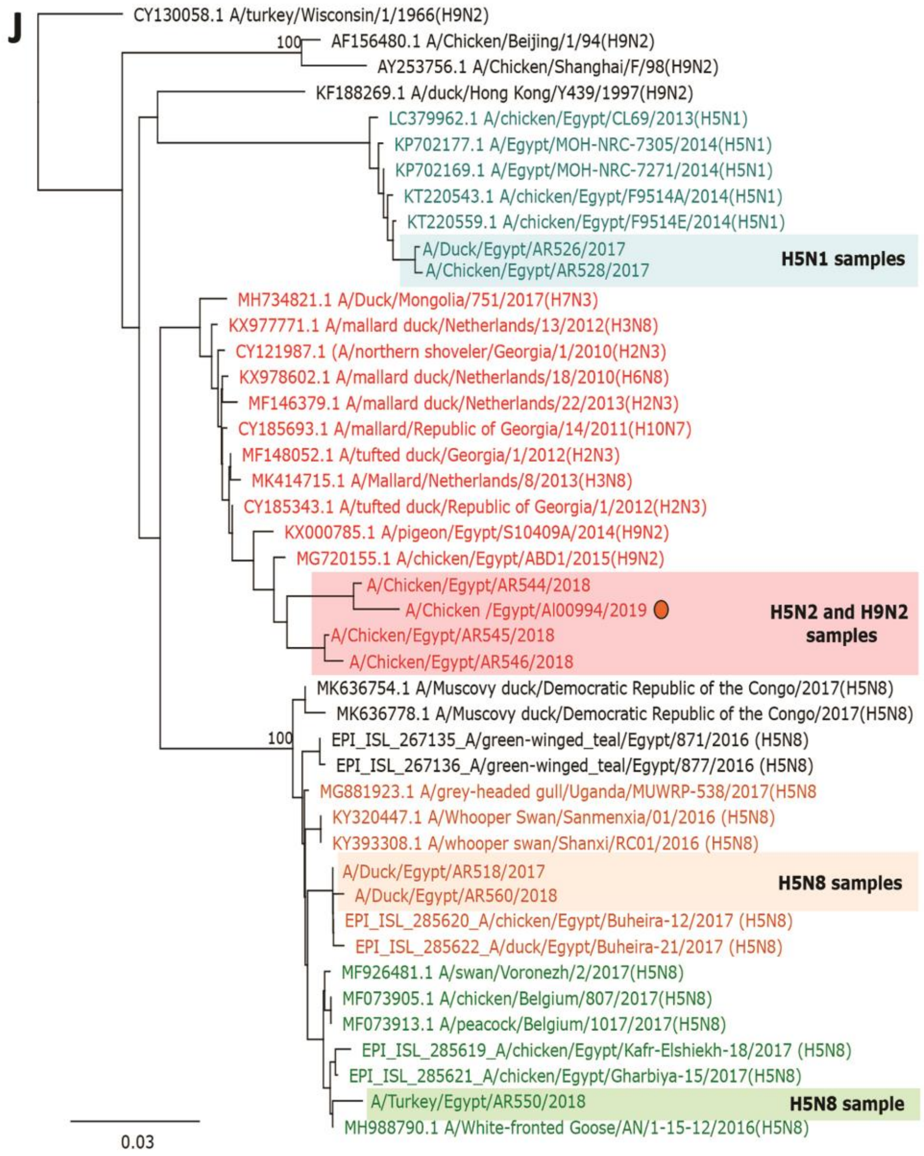

Appendix Figure. Phylogenetic analysis of segments of highly pathogenic avian influenza (HPAI) and low pathogenicity avian influenza (LPAI). Sequence analysis was based on alignment analyses by MAFFTv7.450 embedded in the Geneious software suite, version 11.1.7(https://www.geneious.com) with manual editing. We performed maximum likelihood calculations using PhyML version 3.0 (http://www.atgc-montpellier.fr/phyml) ,we chose the best-fit model according to the Bayesian selection criterion using Model Finder embedded in the Geneious software suite, version 11.1.7https://www.geneious.com). Colors indicate grouping of segment origin according to phylogenetic analyses: highly pathogenic avian influenza virus (HPAIV) H5N1 2.2.1.2 from Egypt (cyan); H9N2 subtype from Egypt circulating in chickens since 2010 (gray); H9N2 subtype from Egypt first detected in pigeons in 2014 (red); PB2 segment most closely related to an H3N6 virus from Bangladesh (purple); PB1 segment most closely related to an H7N7 virus from Georgia (blue); segments most closely related

to H5N8 viruses from China (orange) or Russia (green). Acknowledgments for sequences shared via EpiFlu are given in Appendix Table 2. A) Polymerase basic protein 2 (PB2) of HPAI H5N8, H5N1, and reassortant H5N2, and LPAI H9N2. B) Polymerase basic protein 1 (PB1) of HPAI H5N8, H5N1, and reassortant H5N2 and LPAI H9N2. C) Polymerase acidic protein (PA) of HPAI H5N8, H5N1, and reassortant H5N2, and LPAI H9N2. E) Hemagglutinin (HA _9) of LPAI H9N2. F) Nucleoprotein (NP) of HPAI H5N8, H5N1, and reassortant H5N2, and LPAI H9N2. G) Neuraminidase protein (NA_2) of HPAI reassortant H5N2 and LPAI H9N2. H) Neuraminidase protein (NA_8) of HPAI H5N8. I) Matrix protein (M) of HPAI H5N8, H5N1, and reassortant H5N2, and LPAI H9N2. J) Nonstructural protein (NS) of HPAI H5N8, H5N1, and reassortant H5N2, and LPAI H9N2.
